# Fibula Musculo-Osteocutaneous Flap with Full Thickness of Flexor Hallucis Longus Muscle

**DOI:** 10.29252/wjps.10.1.22

**Published:** 2021-01

**Authors:** Mysore Srinivas Venkatesh, Mohan Kumar Kumaraswamy, Shantha Kumar Shivalingappa, Veena Prabhakar Waiker

**Affiliations:** 1 Department of Plastic Surgery, Ramaiah Medical College and Hospital, Bengaluru, 560040, Bangalore, India

**Keywords:** Fibula flap, Flexor hallucis longus muscle, Arteria peronea magna

## Abstract

**BACKGROUND:**

Fibula flap has been a gold standard method for reconstruction of the mandible. It has been used for reconstruction of maxilla as well as long bones, effectively. Fibula flap in selected cases has been used as a pedicled flap. The flexor hallucis longus muscle has been used to obliterate the dead space during the reconstruction. With a wide range of indications, the use of flexor hallucis muscle has studied for the reconstruction.

**METHODS:**

In a retrospective case record analysis study, 38 subjects were enrolled, included 32 patients with reconstruction of mandible, 1 patient with reconstruction of maxilla, 4 patients with 3 free flaps and 1 pedicled flap with reconstruction of the tibia.

**RESULTS:**

The success rate was 89%, with 4 flap failures. The muscle was used for reconstruction of the tongue, floor of the mouth, antrum, and to cover the fibula graft.

**CONCLUSION:**

Flexor hallucis longus muscle harvested with the flap could decrease the operative time, ease the harvest, and fill the dead space during reconstruction.

## INTRODUCTION

Free Fibula flap was first described by Taylor *et al.*^1^ and is now considered as the gold standard for reconstruction of the mandibular defect.^2^ The fibula osteo-cutaneous flap is used for reconstruction of many anatomical defects. They are used in reconstruction of mandible, maxilla,^3^ clavicle,^4^ long bones (humerus, radius, ulna, femur, tibia),^5^ vertebrae, calcaneum,^6^ etc. The length of the bone that can be harvested, presence of muscle, and large skin paddle that can be harvested have added to the advantages of using fibula flap for reconstruction purposes. 

The fibula flap can be used as a pedicled flap or a free flap. The peroneal vessels supply the flap and on an average measure 2.83 mm in diameter.^7^ Usually, the cutaneous component is supplied by the perforators, from the peroneal vessels. There are numerous perforators along the length of the posterior border of the fibula at an interval of 3-5 cm.

The perforators may travel through either, the flexor hallucis longus, tibialis posterior, soleus muscle^8^ or can be a septo-cutaneous variety. The fibula bone is supplied by the nutrient artery that enters at the middle third and the periosteal vessels. This periosteal supply helps bone perfusion, even after multiple osteotomies. The flexor hallucis longus muscle is predominantly supplied by the peroneal vessels and can be used to obliterate the dead space. Thus, fibula flap has been a versatile flap and we intend to evaluate the flap done at our institute.

## MATERIALS AND METHODS

It is a retrospective case record analysis of patients who underwent fibula osteo-musculo cutaneous flap harvest between June 2015 and December 2019. 38 patients were included where full thickness flexor hallucis longus muscle were harvested in the fibula flap. Institutional ethical committee clearance was obtained for the study. Informed consent has been taken from each patient. The procedures were done under general anaesthesia. Opposite leg was chosen for all cases except for the pedicled flap. 6 cm of fibula were preserved at the upper and lower end. Pre-operative Doppler was used to identify the perforators. 

**Fig. 1:**
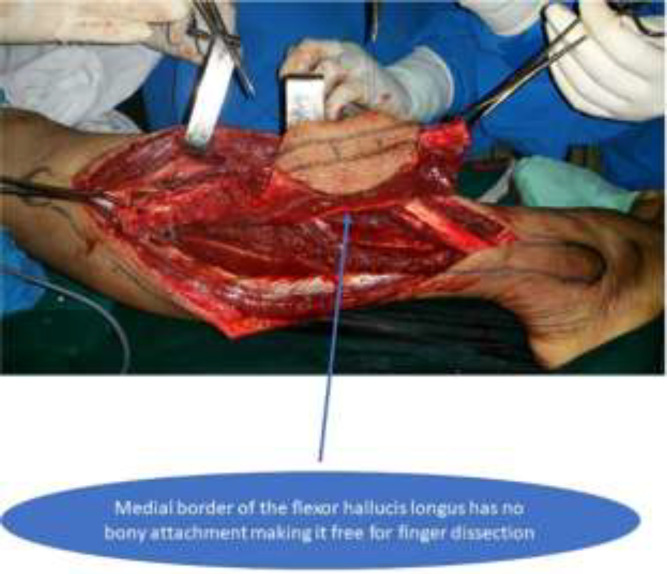
Harvesting the fibular osteo-musculo-cutaneous flap with full thickness of the flexor hallucis longus muscle

**Fig. 2:**
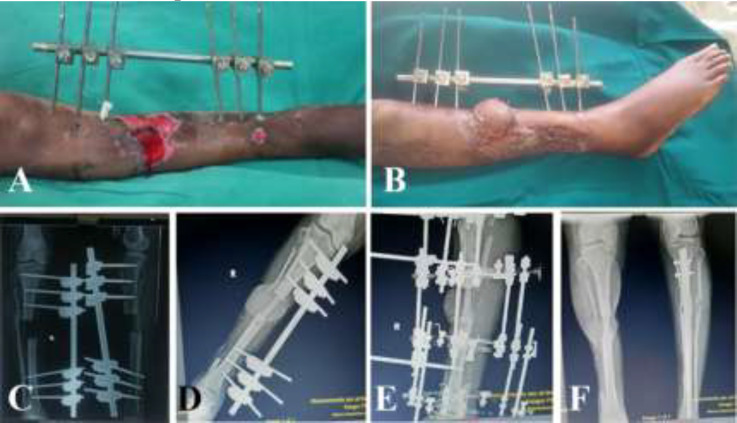
Leg defect after wound optimisation has skin loss with tibial defect. **A:** Early post-operative picture of the settled flap. **B:** X ray of post-tibia debridement showing the bone defect. **C:** X ray immediate post-operative with external fixator. **D:** X ray Ilizaro’s fixator after fibula reconstruction. **E and F:** X ray post-Ilizaro’s fixator removal showing adequate tibialization of fibula

All flaps were harvested by standard anterior approach. Full thickness of the flexor hallucis longus muscle were harvested in the flaps. All three vessels were identified before dividing the peroneal vessels. In case of the pedicled flap, vascular sufficiency of the limb was confirmed on table by clamping the peroneal vessels before division of the vessels. The flexor hallucis muscle was divided at the level of the distal end of the peroneal vessels (Figure 1). The donor site was closed in layers with a drain and covered with split skin graft. The fibula was modelled on a pre-plating technique for the mandible reconstruction. In patients with tibia reconstruction, tibia was reamed and fibular ends were remodelled and were jacked into the tibia, followed by adequate compression. The patient with pedicled fibula transposition, further underwent Ilizaro’s fixation and the fixators were removed after 6 months (Figure 2). All patients undergoing head and neck reconstruction underwent tracheostomy. Heparin 3000-5000 IU was used intravenously before the release of anastomotic clamps. 

This was followed by 40 mg low molecular weight heparin, subcutaneously twice a day for 5 days; 150 mg aspirin tablets, once a day starting on second day for 3 weeks. In patients who had re-exploration for vessel thrombosis, 800 IU/hr heparin infusion was used for 7 days. Patients were allowed as non-weight bearing walking for 3 weeks followed by complete weight bearing after 6 weeks. Patients were followed up for an average of 9 months. In our present series, we had one case of arteria peronea magna. Flap harvest was abandoned and a pectoralis major myo-cutaneous flap was used to cover the defect. The flexor hallucis longus muscle was divided at the lower end of the flap and further dissection was easily done with finger, thus decreasing the time and as well as decreasing the surface area of the raw cut end of the muscle at the donor and the recipient sides. 

We harvested flexor hallucis muscle along with the fibula and used it to obliterate the dead space in the floor of the mouth in case of mandible reconstruction. The mandibular reconstruction was done after oral cancer resection with neck dissection to leave a lot of space in the floor of the mouth. The flexor hallucis longus muscle was used to obliterate this dead space. We used the flexor hallucis longus muscle to obliterate the antrum. The flexor hallucis longus muscle was also used to provide bulk after partial glossectomy. The fibula ends were inserted into the marrow by reaming the tibia and by narrowing the ends of the fibula like a pencil tip.

## RESULTS

Among patients, 27 were male and their age ranged from 29 to 68 years with an average age of 54 years. In one patient, the flap was abandoned after an arteria peronea magna was identified during harvest. Thirty-six patients underwent reconstruction using free vascularised fibula flap and one patient received pedicled fibula flap transposition (Table 1). Thirty-two cases were post-cancer resection reconstruction and 5 patients were for post-trauma reconstruction. We used deltopectoral flap in one case and fascio-cutaneous flap in another one patient along with the fibula flap. Six patients had diabetes; one had neoadjuvant chemotherapy before reconstruction with fibula flap reconstruction. 

Harvest time was between 90 minutes and 200 minutes. Nine cases had single perforator, and 28 had more than one perforator. The reconstructed bone segment ranged from 4 to 15 cm (Table 2). The perforators identified by pre-operative hand held Doppler coincided in 30 cases (81%). The fascio-cutaneous component ranged between 6 and 10 cm in width and from 6 to 15 cm in length. The peroneal vessels were anastomosed end to end to facial artery in 27 cases on the ipsilateral side, and on the contralateral facial artery in 3 cases. It was anastomosed to the inferior thyroid artery in 3 patients. 

For lower limb reconstruction, we anastomosed to anterior tibial vessels in 2 patients, and in one case to the femoral vessels through an interposition vein graft. We used interposition vein graft for artery in 3 patients, and for vein in 4 cases.

**Table 1:** Fibula flap used in different bone reconstruction

**Area of reconstruction**	**Number of cases (%)**
Maxilla	1 (2.7)
Mandible	32 (86.4)
Tibia	4 (10.8) [3 cases free fibula, 1 case of pedicled fibula]

**Table 2:** Mandible reconstruction with number of patients

**Mandible segment reconstructed**	**Number of cases (%)**
Lateral segment alone (from Para symphysis to ramus)	20 (62.5)
Lateral and central segment	11 (34.4)
Both lateral and central segment (from ramus on one side to ramus on the other)	1 (3.1)

Regarding complications, re-exploration was necessary in 6 subjects, venous thrombosis in 5 cases and arterial occlusion in one patient. We were able to salvage the flap in 2 cases, and lost the flap in 4 patients and the flap success rate was 89%. Of the 4 flaps which were lost, two were used for mandible reconstruction and the other two were used for tibia reconstruction. 

We reconstructed with pectoralis major myo-cutaneous flap in 2 patients suffering from buccal cancers. Out of the two patients where fibula was used for tibia reconstruction, one had amputation, and the other had local flap cover and Illizaro’s bone transport. Donor site minor loss of skin graft was noted in 4 patients (10.8%) who were treated with regular dressings, and major skin graft loss in 2 patients (5.4%) who required skin grafting. One patient had deep vein thrombosis at the femoral and the external iliac veins, 4 weeks after surgery that was treated with anticoagulants. Three patients complained of hair growth inside the mouth who did not receive for post-excision radiotherapy. One patient with tibia reconstruction developed osteomyelitis after 1 year and his limb was not salvageable and underwent amputation. 

**Fig. 3:**
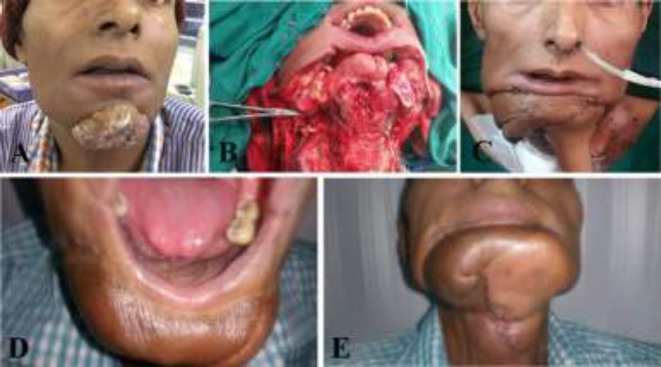
Pre-operative picture of buccal cancer with skin involvement. **A: **Post-excission defect with loss of central segment of mandible, skin and mucosa. **B and C:** Early post-operative picture of free fibula and deltopectoral flap. **D and E:** Late post-operative picture after division of deltopectoral flap

The preoperative Doppler scan mapping was used to map the perforators. The flaps that were harvested were based on middle and the lower third of the leg. The flaps had perforators in the middle third of the leg in 70% cases. In our series, we had one perforator in 24% cases and we did not encounter any flap partial flap loss in the present series. All our flaps in the present series were type A. In one case, the flap was harvested with a single perforator. There was vascular compromise with complete inset. The flap perfusion was good with partial inset, but there was exposure of the mandibular plate. a deltopectoral flap was used to cover the hardware (Figure 3).

**Fig. 4:**
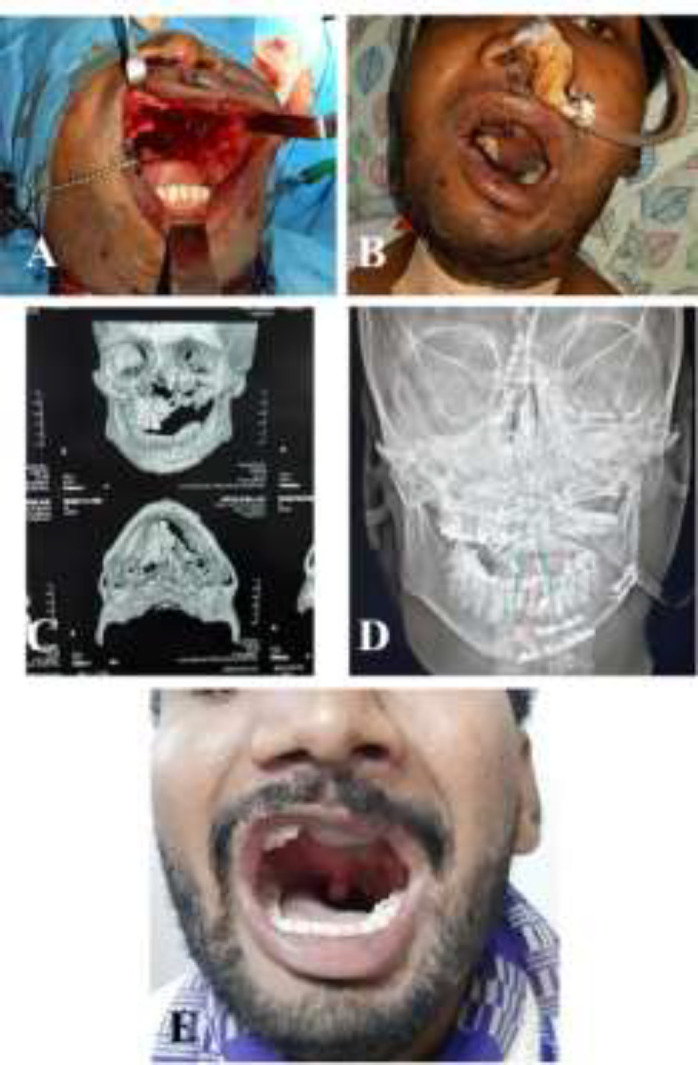
**A: **Post traumatic defect in the maxilla with loss of anterior wall in maxilla, alveolus, and more than half of the palate. **B:** Early post-operative picture of free fibula osteomusculocutaneous flap with flexor hallucis longus used to fill the antrum. **C:** Pre-operative CT scan image showing the defect. **D:** Post-operative X ray film showing the fibula. **E:** Late post-operative photo showing flap well settled

In one case with defect of the lower limb, where free fibula was used to reconstruct the tibia, the skin was inadequate to cover the ankle. We had a choice to cover with a second free flap, but we used an inferiorly based fascio-cutaneous flap. The fibula flap was harvested along with layer of muscle to avoid bearing the periosteum. The tibialis posterior, flexor hallucis longus, and soleus muscle of varying thickness were harvested with the fibula flap. The ease of harvest and the lesser time taken to harvest favoured the harvest of muscle with the flap. In one case of post-traumatic maxilla reconstruction, there was loss of alveolus, and anterior wall of the maxilla (Figure 4). 

In the lower limb reconstruction, the cutaneous part was short in covering the fibula on one side. This was covered with the flexor hallucis longus muscle and the muscle was covered with the skin graft. We did not have any complications that are documented for the use of muscle sparing flap harvest. We reconstructed tibia with fibula in 4 patients. The two patients did not have any fractures on follow up, even after weight bearing mobilisation. We illustrated wound healing problems at the donor site in 16% cases. There were no hematomas between the muscles. The patients did not feel any difficulties in their regular activities too. 

The donor site was covered with skin graft in all cases. The skin graft loss which required repeat skin grafting was 5.4%. The patients did not complain regarding the movement of the great toe. Vein thrombosis, though the patient was on prophylactic dose of tablet aspirin.

## Discussion

After Taylor *et al.*^9^ described the free fibula, and the skin flap for monitoring was explained by Yoshimura *et al.*^10^ The advantages of fibula flap used for mandible reconstruction are better aesthetics, the long available bone, a reasonable shape, the ability to be shaped well to the recipient defect, good calibre vessels and being long enough to reach the neck. The free fibula flap used in reconstruction of the maxilla provides bone and muscle to fill the antrum and the skin. The fibula is straight and long appears to be the distinct advantage of using for reconstruction of long bones.

The tibio-peroneal trunk is a branch of the popliteal artery and is divided into peroneal artery and the posterior tibial artery. Usually, the posterior tibial artery is the dominant supply to the foot. Sometimes, the peroneal vessel becomes the dominant supply to the foot, which is referred as an arteria peronea magna. The arteria peronea magna or the dominant peroneal artery is found from 5.2% to 10% of cases.^11^^,^^12^ The best way to detect the anatomical variation pre-operatively is getting a pre-operative CT angiogram from the leg. However, Alolabi *et al. *concluded that, there is low evidence to suggest the need for routine pre-operative angiogram before harvesting the fibula flap.^13^

If we do not have a pre-operative CT angiogram, we need to be careful. Just before dividing the peroneal vessels, if we identify the posterior tibial vessel of adequate size supplying the foot, we can avoid the mishap of stealing a major blood supply to the foot. In our series, we have come across an arteria peronea magna, and we have abandoned harvest of the flap. If the surgeons do not recognise it and leads to vascular compromise, it may need an interposition vein graft to establish the vascularity.^14^

The preoperative Doppler scan mapping was used to map the perforators. The flaps that were harvested were based on middle and the lower third of the leg. The flaps had perforators in the middle third of the leg in 70% cases. Heitmann *et al.* have also recorded maximum number of perforators in the middle third of the leg.^15^ In our series, we had one perforator in 24% cases and we did not encounter any flap partial flap loss in the present series. All our flaps in the present series were type A identical to Prabha *et al.,*^8^ where the skin paddle was supplied from the peroneal system alone. 

Ideally reconstructing with a single flap would be the best option. Sometimes the fibula flap alone may not be adequate to reconstruct the large composite defect of skin and mucosa. In some centres, to decrease the operative time, the flap is harvested simultaneously along with the tumour excision. In such situations, reconstructive surgeons prefer to harvest a marginally bigger flap and trim it during inset. There may be technical difficulties during inset wherein, a particular position of the flap, especially when, it is folded, it may compromise the vascularity of the flap. In such cases, the flap may be insufficient and we may need to use a second flap.^16^

The second flap could be a second free flap or a second pedicled flap as an anterolateral thigh flap, pectoralis major myocutaneous flap, and deltopectoral flap. Using a pedicled flap as a second flap would decrease the operative time, morbidity, and further it is easy to harvest.^17^ However, Prabha *et al.* were able to cover all defects with a bi-paddled fibula flap without the need for a second flap.^18^ In our series, in one case, we could harvest the flap with a single perforator. There was vascular compromise with complete inset, probably due to stretch on the perforator. The flap perfusion was good with partial inset, but there was exposure of the mandibular plate. So, we had to use a deltopectoral flap to cover the hardware.

In one case with defect of the lower limb, where free fibula was used to reconstruct the tibia, the skin was inadequate to cover the ankle. We had a choice to cover with a second free flap, but we used an inferiorly based fascio-cutaneous flap. The fibula flap was harvested along with thin layer of muscle to avoid bearing the periosteum. The tibialis posterior, flexor hallucis longus, soleus muscle of varying thickness was harvested with the fibula flap. Some authors argued for harvesting muscle to fill the dead space in the floor of the mouth,^18^ and antrum, after partial calcanectomy.^6^

In one case of post-traumatic maxilla reconstruction, there was loss of alveolus, and anterior wall of the maxilla. We used the flexor hallucis longus muscle to obliterate the antrum and to provide bulk after partial glossectomy. In the lower limb reconstruction, the cutaneous part was short in covering the fibula on one side. This was covered with the flexor hallucis longus muscle and the muscle was covered with the skin graft. However, Roger *et al.* recommended use of muscle sparing technique leaving a thin rim of muscle. The advantages include a decreased donor site dead space, ease of osteotomies, and decrease in hematoma at the donor and the recipient site.^19^

Incidence of lower limb defects with bone and skin loss after road traffic accidents is very common in our country based on the high vehicular density and a large number of two- wheelers. If less than 6 cm of the bone defect is present, Ilizaro’s bone transport is a common method to be used for reconstruction. If the defect is longer than 6 cm, vascularised bone graft is a better choice for reconstruction. Because the vascularised bone preserves the function of the osteoblasts and osteoclasts, consolidation and remodelling are incorporated faster and more efficiently.^20^^,^^21^

Fibula bone is straight and long that suits the reconstruction of the long bone. Miguel de la Parra-Marquez *et al.* are also with the opinion that the advantage of using a vascularised fibula is the availability of the length, its shape, and the minimal donor site deformity.^22^ In the present series, we have reconstructed tibia with fibula in 4 patients. The fibula ends were inserted into the marrow by reaming the tibia and by narrowing the ends of the fibula like a pencil tip. This would increase the contact between the bones. This with, adequate compression at the fracture, would help in early fracture healing. The tibialization of the fibula was helped by improved contact and the compression between the bones. 

Thus, in our series, the patients did not have any fractures on follow up, even after weight bearing mobilization. In lower limb reconstructions, where there is a need for reconstruction of long bones and also strength of weight bearing, modified Capanna’s technique has been used. Here, vascularised fibula with an allograft has been used.^23^ The authors have explained that the allograft would help in providing the strength until the fibula attains the size of the recipient bone. If we do not have an option of using an allograft, use of doble barrel fibula is recommended which would add strength to the reconstructed bone.^24^^,^^25^

The complications usually seen after fibula harvest could be skin graft loss, sensory problems, weakness of the toes, wound healing, and hematoma. In our series, we had wound healing problems at the donor site in 16% cases. The hematomas under the skin graft have led to the skin graft loss. However, there were no hematomas between the muscles. The patients did not feel any difficulties in their regular activities too. The fibula flap donor site can be closed primarily if the skin paddle harvested has 4 cm in breadth, or it can be covered with skin graft. We had to cover the donor site with skin graft in all cases, since the breadth was more than 4 cm. The skin graft loss, which required repeat skin grafting, was 5.4%, which was comparable to other authors.^26^

The patients did not complain regarding the movement of the great toe. One to 6% was the incidence of deep vein thrombosis in free flap harvest. The surgical trauma to the leg and the decreased mobility after the surgery could cause the deep vein thrombosis. One patient, in our series had deep vein thrombosis, though the patient was on prophylactic dose of tablet aspirin. Though aspirin prophylaxis decreases the chance of deep vein thrombosis, there is still a risk. Mhamad *et al.* documented that patients with knee and hip surgeries had venous thrombosis at a rate of 0.9%, even with prophylactic low dose aspirin. Encouraging the patients to move, physiotherapy may further help in decreasing the deep vein thrombosis.^27^

## CONCLUSION

Fibula flap is a versatile flap that can be used in reconstruction of mandible, maxilla, long bones. Pre-operative CT angiogram would help identifying the arteria peronea magna. Use of clamps on the peroneal vessels and identifying all the three major vessels would confirm the adequacy of the vascularity of the foot in the absence of CT angiogram. The flexor hallucis longus muscle harvest would decrease the operative time, the ease to harvest of flap and to fill the dead spaces in the recipient site.
